# Fabrication of a Miniature Paper-Based Electroosmotic Actuator

**DOI:** 10.3390/polym8110400

**Published:** 2016-11-15

**Authors:** Deepa Sritharan, Elisabeth Smela

**Affiliations:** 1 Department of Mechanical Engineering, University of Maryland, College Park, MD 20742, USA; deepey@umd.edu; 2 Institute for Systems Research, University of Maryland, College Park, MD 20742, USA

**Keywords:** electrokinetic flow, compliant, additive manufacturing, biomimetic, artificial muscle, nastic, smart material, electro-active polymer device

## Abstract

A voltage-controlled hydraulic actuator is presented that employs electroosmotic fluid flow (EOF) in paper microchannels within an elastomeric structure. The microfluidic device was fabricated using a new benchtop lamination process. Flexible embedded electrodes were formed from a conductive carbon-silicone composite. The pores in the layer of paper placed between the electrodes served as the microchannels for EOF, and the pumping fluid was propylene carbonate. A sealed fluid-filled chamber was formed by film-casting silicone to lay an actuating membrane over the pumping liquid. Hydraulic force generated by EOF caused the membrane to bulge by hundreds of micrometers within fractions of a second. Potential applications of these actuators include soft robots and biomedical devices.

## 1. Introduction

Soft actuators are able to stretch, twist, squeeze through tight spaces, and gently grip fragile objects (see reviews [1,2]) because they are constructed using deformable materials. Motors are not used for actuation. Instead, force generation is achieved by mechanisms such as electrostatics, phase change, and mass transport, among others. Electroactive and stimuli-responsive polymer actuators span a wide range of materials and performance (see recent reviews [1,2,3] and conference proceedings [4,5,6]). The electroosmotic actuator presented here makes use of a different actuation mechanism, based on microfluidics [7,8].

Fluidic actuators employ pressurized fluids, either gases (pneumatic) or liquids (hydraulic) [9], to generate motion by causing inflatable or deformable fluid-filled chambers to change shape. Fluidic actuators are capable of large movements and can achieve high work densities. The earliest and most extensively studied pneumatic soft actuators are McKibben muscles [10], which are composed of an elastic tube surrounded by a sleeve, and which contract with pressure [11,12]. Recently, soft robots have been demonstrated that contain multiple fluidic channels embedded within an elastomer [13] to provide more complex motions such as limb-like motion [14], crawling [15], and grasping [16]. Curving, for example, can be achieved with fluidic channels separated by a bendable, but not stretchable, layer [16], which is often made of fabric, paper, or a stiffer polymer. Both pneumatics [17,18,19] and hydraulics [20] have been used to realize soft robots. Pneumatic actuators are capable of rapid movements, enabling motions such as jumping [21]. Hydraulic actuators are suitable for larger loads because liquids are incompressible, resulting in a higher strength-to-weight ratio, lower pressure losses, and good power transmission. There are relatively few miniature hydraulic actuators due, at least in part, to the difficulty of constructing leak-proof devices at cm-scales [22].

Fluidic actuators are usually operating with pressure-driven flow by connecting them, via tubing, to a compressed gas tank (pneumatic) or an external pump (pneumatic or hydraulic), limiting the system’s motion. Eliminating tethers would be advantageous to achieving stand-alone robots, and would facilitate miniaturization and integration with other systems. Untethered force delivery in pneumatic systems has been achieved by generating gases within the actuator by decomposition of the liquid propellant via peroxide reactions [14,23], combustion [24], thermal liquid-vapor phase change [25,26], or electrolysis [27,28]. Regrettably, these devices have short lifetimes (seconds to minutes) because of reactant depletion. Untethered hydraulic actuation has not been demonstrated, but applying an electric potential to generate electrokinetic flow [29] provides a possible means for that. This would theoretically allow fine control of actuator motion using electronic circuits, while achieving longer device operation because no decomposition occurs.

Here, we describe a fabrication process to create fully-sealed actuators via layer-by-layer assembly of flexible materials: silicone and paper. The paper is sandwiched between elastomeric electrodes, and the pores in the paper serve as the fluidic channels. The process includes a tubing-free method to fill the device with the pumping liquid and then encapsulate it. Electrohydraulic fluid flow occurs upon applying an electric field, which inflates the membrane. Actuator characterization shows that large displacement, reasonable force, and good speed can be achieved by electrohydraulic force transmission.

## 2. Materials and Methods

### 2.1. Materials

The microchannel layer consisted of Whatman cellulose filter paper (No. 2, qualitative, pore size 8 μm, particle retention efficiency 98%, thickness 190 μm).

The pumping liquid for electroosmosis was propylene carbonate (PC) (anhydrous 99.7%, Sigma-Aldrich, St. Louis, MO, USA, 310328). The PC was stored in a glove box with an argon atmosphere because exposure to ambient moisture causes irreversible chemical degradation [8]. The device fabrication and experiments were, however, performed under atmospheric conditions, not in a glove box.

Three platinum-cure silicone elastomers were used: polydimethylsiloxane (PDMS) (Dow Corning, Midland, MI, USA, Sylgard 184, 10:1 base:curing agent) and Ecoflex 00-50 and 00-30 (Smooth-On Inc., Macungie, PA, USA, 1:1 parts A:B). PDMS is transparent, has a durometer hardness of 43 Shore A (at 10:1), and has an ultimate strain of 150% [30]. Ecoflex 00-50 is translucent, has a durometer hardness of Shore 00-50, and has an ultimate strain of 980% [31]. All three were cured at 65 °C in an oven for 1–2 h, depending on thickness. Spin-coated layers of PDMS (200 rpm, 60 s, 50 rpm/s ramp) were allowed to rest at room temperature for at least one hour to allow air bubbles to escape before curing.

Mold release (Mann Ease Release 200) was applied to glass substrates (slides 5 × 7.5 cm^2^) to allow removal of the elastomers. Mold release was not required when using transparency sheet substrates because the silicones readily peeled from the plastic. In fact, when patterning the elastomers with a cutting machine, mold release treatment of the transparency caused the films to delaminate and get damaged by the blade.

### 2.2. Patterning

Filter paper was patterned using a computer-controlled electronic plotter cutter (Cricut Explorer, paper setting). The paper was adhered to a cutting mat. To prevent damage to the paper during release after cutting, the tackiness of the mat adhesive was reduced by dabbing it lightly with cotton fabric. The final shapes were released by sliding a razor blade beneath them; this avoided paper curling, which occurred if the paper was peeled off the mat.

Electrodes were patterned either by cutting (manually with a razor blade or with the Cricut Explorer, pressure 129, “aluminum foil” setting), punching (Harris Unicore tissue coring tool), or printing through stencils. Stencils were formed by cutting (Cricut Explorer, cardstock setting) multiple layers of tape (3M St. Paul, MN, USA, Scotch blue masking #2080EL, thickness *t* = 0.0038″ = 90 μm): 5 for the device layer (*t* = 483 μm) and 4 for the reservoir. The cut pressure was adjusted so that the blade did not score the underlying substrate; score marks were replicated as ridges when the electrodes were embedded in PDMS, lowering the quality of plasma bonding and leading to leaks during operation. 

Film roughness was determined with a mechanical profilometer (Tencor, Manassas, VA, USA, AlphaStep 200). Thickness was measured with calipers or a ruler.

### 2.3. Electrode Materials

Electrodes were composed of 14 wt % carbon black (40 nm, Alfa Aesar, Haverhill, MA, USA, 39724) mixed into the silicone elastomer (PDMS or Ecoflex), prior to curing. Suspensions were prepared in a 20 mL glass vial, starting with adding 0.07 mg of carbon black to the pre-polymer (4.5 g of PDMS base or 2.5 g of Ecoflex part A). After manual stirring, the suspension was vortex mixed (Fisher Scientific digital vortex mixer, 60 s, 3000 rpm) and sonicated in a water bath (40 kHz, 20 min) at room temperature. Curing agent was added to the suspension (0.45 g for PDMS or 2.5 g for Ecoflex B), which was again stirred, vortexed, and sonicated (1 min).

In addition, a faster mixing technique was tested (5 min total preparation time vs. 25 min) in which carbon black, pre-polymer, and curing agent were added all at once to the mixing container of a planetary centrifugal mixer (Thinky, Tokyo, Japan, Mixer ARE-310) used in standard mode (30 s mixing at 2000 rpm, 30 s de-foaming at 2200 rpm). The mixture was scraped off the sides of the container, stirred with a dowel, and placed back into the mixer again. This was repeated four times, for a total time of 4 min. in the mixer. Both mixing methods resulted in smooth pastes with the consistency of toothpaste and similar spreadability. The paste adhered to the blade.

PDMS/carbon (C-PDMS) paste was applied to cover the stencil and doctor bladed (6 passes with a razor blade) to ensure a uniform layer with a thickness determined by the stencil height. The stencil was peeled off, and the C-PDMS cured. Doctor-blading yielded a top surface with high roughness (≥130 μm, exceeding what could be measured by profilometry), and a bottom surface, facing the glass, that was smooth (roughness ≤2 μm) and glossy. Low surface roughness is required for successful plasma bonding. Surface roughness may also alter the structure of the electrical double layer at the electrode-electrolyte interface [32,33,34], which would lead to variability in actuator performance. Smooth, homogeneous electrode and channel surfaces enable better control and predictability of EO flow [34]. Further information regarding the characterization of the electrode materials is provided in the Supporting Information.

### 2.4. Plasma Bonding

Plasma bonding was used to adhere the device layers. Surfaces were treated in oxygen plasma (Branson 3000 Barrel Resist Stripper, 20 s, 50 W, 1 Torr) and immediately brought into contact. The process required surfaces to be clean, flat, minimally rough, and free of defects such as holes, scratches, and particulates. Fingerprints, dust, moisture, and impressions formed from scratches impaired the bonding.

### 2.5. Actuator Performance Testing

Membrane deflections and forces were measured using a force/strain transducer (Aurora Scientific, Aurora, ON, Canada, 300B), the former in isotonic mode (constant force) and the latter in isometric mode (constant position). The transducer was interfaced to a computer via a data acquisition card (National Instruments), and data were recorded using a custom LabVIEW program.

The actuator was fixed to a glass slide, membrane-side-up. The carbon-elastomer electrodes were connected to the power supply (HV Rack, Ultravolt) with toothless alligator clips. A pressure-distribution plate, consisting of a piece of transparency film (5 × 5 mm^2^), was manually centered on top of the membrane, and the lever arm of the transducer was positioned to rest in the middle of it (Figure 2). The plate was coated with mold release to minimize stiction to the membrane.

## 3. Device Design

The goal of the present work was to create miniature hydraulic soft actuators: self-contained, untethered, high-force devices that are voltage controlled. The design of the actuators was inspired by plant motion. Although plants lack muscles, they can generate a variety of movements, both slow and fast, by pumping water in and out of their cells via osmosis [35]. Mimicking the plant cell design, our actuators comprised an elastomer with embedded fluid-filled reservoirs connected by microchannels. Application of an electric field across the microchannels induced electroosmotic flow of the liquid from one reservoir to another, causing deformation of the surrounding elastomer.

### 3.1. Pumping Mechanism

A variety of flow effects occur when an electric field is applied to a liquid, but the electrokinetic effects that have been used for generating bulk flow are electroconjugate flow, electrowetting, and electroosmosis. In electroconjugate flow, fluid jets are generated with dielectric liquids [36,37,38,39,40]. Electrowetting actuators employ flow driven by applying a voltage to the channel wall [41]. Our research focused on employing electroosmosis, also a surface phenomenon like electrowetting but distinguished by the fact that flow is initiated by an electric potential applied over the liquid, instead of the contacting solid.

The surfaces of solid objects in contact with a fluid have a net charge [42]. Mobile counter-ions (ions in the fluid with opposite charge to the surface) are attracted to the surface to maintain electrical neutrality, resulting in a so-called electric double layer, one side of which is positive and the other of which is negative; the potential difference is called the zeta potential. The bulk of the fluid remains charge neutral. When an external electric field is applied along a fluid-filled channel, the mobile charges in the double layer are induced to move. The direction of fluid flow depends on the sign of the surface charge. This counter-ion motion along the walls transfers momentum to the remaining fluid, resulting in bulk flow with a nearly flat velocity profile. Microfluidic devices employ electroosmosis to achieve fast, pulseless pumping at high forces [43,44,45,46,47,48,49,50,51,52,53,54].

Electroosmotic flow in *N* channels of length *L*, cross-sectional area *A*, and depth *d*, filled with a liquid having permittivity *ε* and viscosity *η*, and employing a channel material giving a zeta potential *ζ* is believed to be described by [55]:

(1) $$\text{EO flow rate}=NA\frac{\varepsilon \zeta E}{\eta },$$

(2) $$\text{EO pressure}=\frac{12\varepsilon \zeta EL}{d^{2}},\;\text{in rectangular channels (depth << width)}.$$

For high force and fast EOF, the device requires: (a) a large number of small channels with high surface charge; and (b) a low viscosity and a liquid with high permittivity.

In addition, the liquid must exhibit electrochemical stability. Typically, water is used in EO pumps due to its high polarity, but in water, electrolysis causes depletion of the pumping liquid, changes in pH, and evolution of gas bubbles, resulting in irreproducible pumping, low actuation pressure, and device failure. In this paper, we used propylene carbonate (PC) as the pumping liquid because it exhibits bubble-free operation up to kV [8,56]. The zeta potential for both water and PC in PDMS is negative [8]: fluid moves from the positive electrode toward the negative electrode, indicating that the mobile charges at the walls are positive (cations).

### 3.2. Paper Microchannels

The first electroosmotic actuators we reported [7,8] were fabricated entirely from PDMS by soft lithography using a microfabricated SU-8 mold. They contained nine rectangular channels each 1 cm long, 150 μm wide, and 25 [7] or 40 μm deep [8].

While forming microchannels by soft lithography is well suited for the creation of lab-on-a-chip devices, for EO a large number of small microchannels is needed to produce a significant flow rate. Equation (1) shows that flow rate depends on the total cross-sectional area *NA*, and Equation (2) that force depends inversely on the square of the channel depth *d*. One approach to increasing the number of pumping channels and reducing their diameter is to pack conventional microfluidic channels with beads [48] or porous monoliths [47,48,57,58,59,60]. Another approach is to employ pre-formed microporous structures, such as mullite [50], porous glass frits [44], or porous elastomeric sponges [56].

**Figure 1:**
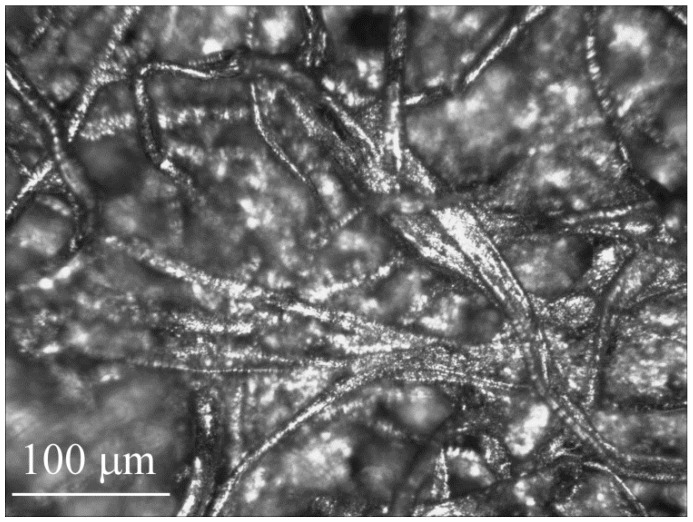
Micrograph of the surface of Whatman 2 filter paper, coated with platinum for enhanced contrast to permit visualization of the fiber structure.

Here, we employ the second approach, with paper as the microchannel layer, since it is highly porous and readily absorbs liquid between the cellulose fibers. Figure 1 shows the microstructure of the filter paper used in this work. It consists of interwoven cellulose fibers, touching in places and separated by a range of relatively large gaps in others.

There is extensive literature on paper-based microfluidics, as reviewed in [61,62], and electroosmosis in paper has been observed as a side effect during electro-chromatography [63], but paper has yet to be employed for electroosmotic pumping. Paper microchannels, unlike those made of silicone, wick fluids such as water and PC, thereby facilitating bubble-free device filling. PC did not appear to affect the paper, which maintained its shape after soaking in PC. While paper exposed to water caused swelling and warping, this was absent in PC. No visual deterioration was observed in paper that had been stored in PC for over two years at atmospheric conditions.

There are other properties of paper that make it attractive for the creation of soft actuators: it can be folded, bent, cut, and stacked to build 3D structures. It is also readily available, inexpensive, and reasonably mechanically robust. For microfluidics, papers with good wet strength (a measure of the ability to withstand rupture when the paper is wet) are filter paper and tissue paper.

### 3.3. Device Design

**Figure 2:**
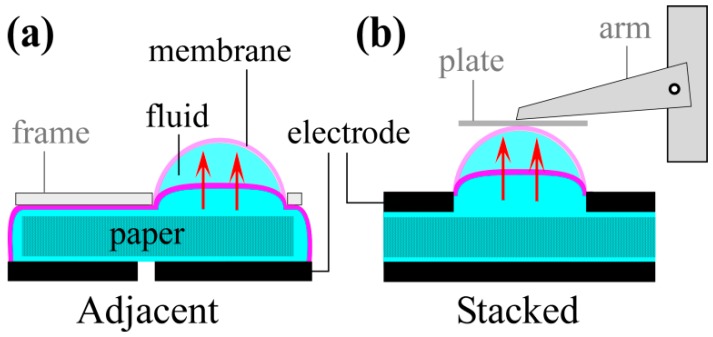
Device designs with (**a**) adjacent and (**b**) stacked electrodes. A paper microchannel layer is positioned between the electrodes. Fluid pumped through the paper inflates an elastomeric membrane. Force and deflection are measured using a transducer, the arm of which rests on a pressure-distributing plate placed on the membrane.

The device essentially comprises fluid-filled paper between a pair of electrodes covered by an elastic membrane (Figure 2). Applying a voltage across the electrodes leads to EO pumping within the paper, in some cases along with other electrokinetic fluid motions, as discussed below. Transferring the fluid toward the membrane leads to its bulging outward. Elsewhere, not shown in the figure, the structure collapses to maintain constant volume. The membrane is thin and elastic to allow large displacements. Bulging membrane designs are used in valves and tactile displays. Pumping fluid between reservoirs within an elastomeric material, rather than under a membrane, would result in structural deformations.

Two configurations were investigated. In the “adjacent” layout (Figure 2a), electrodes were situated side-by-side in a planar arrangement, with the paper on top of them. In the “stacked” layout (Figure 2b), electrodes faced each other with the filter paper sandwiched between them. In both cases, the electrodes were placed as close together as feasible: from Equations (1) and (2), both flow rate and pressure scale with the electric field, so decreasing the electrode separation lowers the required voltage. The planar configuration resulted in thinner devices, allowing them to be smaller and more bendable. Fabrication was also simpler, as discussed below, because it entailed fewer steps and no plasma bonding, and because either PDMS or Ecoflex could be used throughout the device. However, the smallest distance between electrodes was 0.5 mm, set by the ability of the cutting machine. In the stacked configuration, the electrode spacing was closer, since it was set by the paper thickness and did not depend on patterning.

## 4. Fabrication

**Figure 3:**
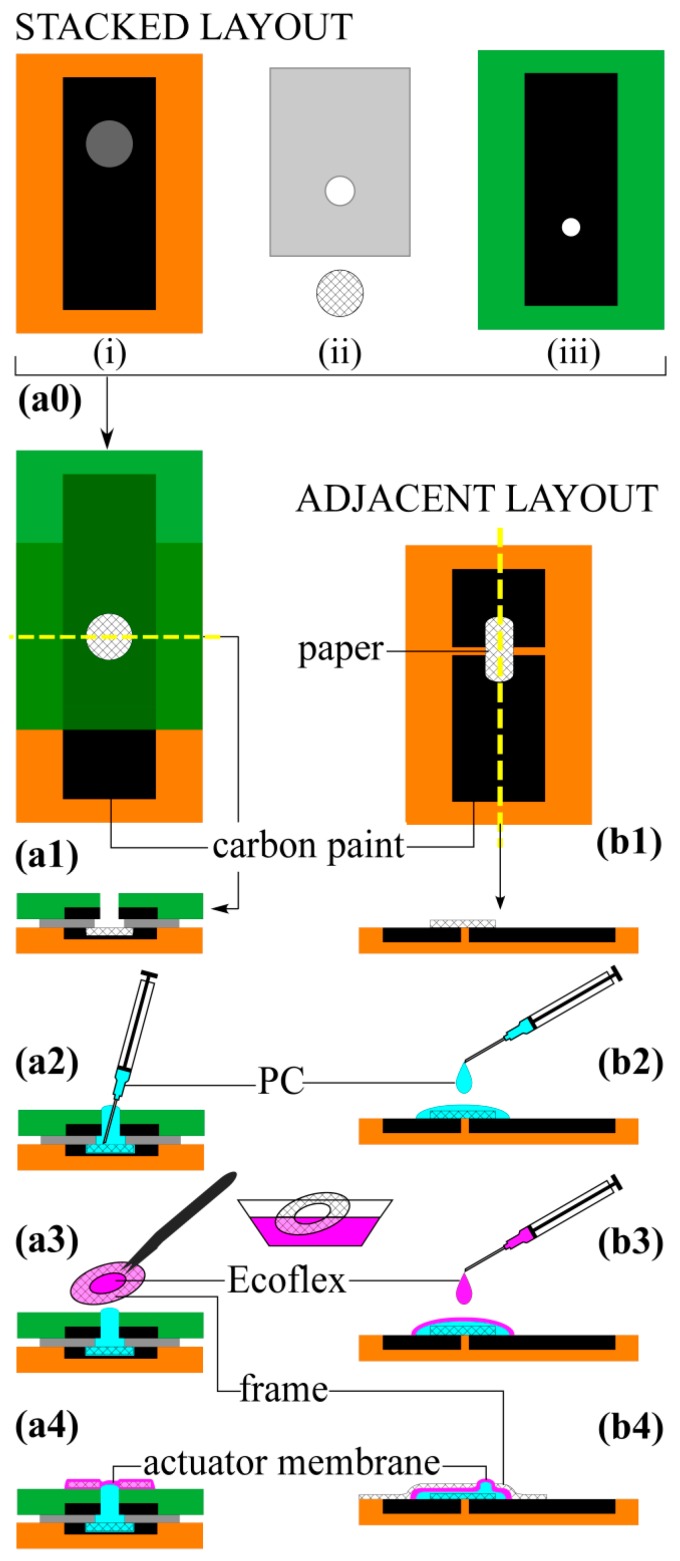
Fabrication process for actuators with stacked (**a0–a4**) and adjacent (**b1–b4**) electrodes. (**a0**) The stacked layout began with preparing (**i**) a bottom electrode layer (orange) with a reservoir; (**ii**) an insulating layer (gray) and a paper disk; and (**iii**) a top electrode layer (green). The electrode layers were patterned by doctor-blading C-PDMS over a stencil, curing, and embedding in PDMS. Holes were punched in the upper two layers to form the fluid port. (**a1**,**b1**) Assembly. (**a1**) The components of the stacked layout were plasma treated, aligned, and bonded. Overhead and cross-sectional views are shown; the section is indicated by the dashed line; (**b1**) In the adjacent layout the electrodes were patterned using a cutting machine and embedded in PDMS. The paper was placed over the electrodes. (**a2**,**b2**) Addition of pumping liquid. (**a2**) Liquid was added via the inlet hole; (**b2**) Liquid was dispensed onto the paper. (**a3**,**b3**) Encapsulation. (**a3**) A paper frame was dipped in uncured Ecoflex and laid over the inlet port; (**b3**) Ecoflex was dispensed directly on the PC droplet. (**a4**,**b4**) Completed devices. Frames defined the deflecting regions.

Because the goal was to create an electrically-controlled, fully flexible actuator, every part of the device needed to be compliant. Two elastomers were used in this work: PDMS for the structural components and the more flexible Ecoflex 00-50 for the actuating membrane. Thin film metal electrodes with serpentine shapes allow stretching [64], but they degrade under EO. Stretchable electrodes can also be made from composites containing conductive nanoparticles, such as carbon black (CB) [65,66] or exfoliated graphite (EG) [67], within an elastomeric host, but they have not previously been used for EO. Elastomer/EG composites are more stretchable, but CB composites were chosen for this work because the CB mixed more uniformly into the elastomers and large strains were not required. The other materials used in the devices were paper (the microchannel layer), which is bendable but not stretchable, and liquid (the PC pump fluid). The fabrication sequences (Figure 3) for the two designs differed substantially, so they are described separately.

The fabrication method for the carbon-based electrodes required some development, as described in the Supporting Information. A volatile solvent, such as hexane, is typically used to lower the viscosity of the uncured elastomer/carbon mixture, but this negatively affects the cured film by lowering its conductivity, making it brittle, and producing small holes [68]. A solvent can also limit the substrate materials on which the electrodes can be applied. We investigated the effect of solvent on resistance and film quality and concluded that electrodes should be formed without hexane. In addition, PDMS and Ecoflex were compared as host polymers. Ecoflex outperformed PDMS, but we used PDMS for the electrodes because of one critical step in the fabrication process: oxygen plasma bonding of the layers to create thin devices. Plasma surface treatment was ineffective with Ecoflex.

### 4.1. Adjacent Layout

In the adjacent layout, the silicone used for device fabrication could be either PDMS or Ecoflex.

The base layer, on which the rest of the device was constructed, consisted of two electrodes embedded in a thicker layer of silicone (Figure 3(b1)). A large-area film of C-silicone (*t* = 0.5 mm) was formed on a plastic sheet using a stencil, the two electrodes were cut from that, and the rest of the film was peeled away. Silicone was cast over the electrodes (*t* = 1 mm) and cured, and this base layer was peeled off the substrate. The paper microchannel layer was cut into a rounded rectangle (1.5 cm × 1 cm) and placed across the electrodes. The pumping liquid, PC, was dispensed onto the paper using a syringe (0.1 mL, 30 gauge needle) until the paper was saturated; wicking action filled the microchannels (Figure 3(b2)). Excess PC formed a droplet around the paper and held its shape due to surface tension. The PC was encapsulated by dispensing liquid silicone (0.3 mL) around the base of the PC drop, at the liquid–substrate interface, until the silicone spread from the periphery of the droplet towards the top to form a thin film (Figure 3(b3)). The membrane was cured in an oven (65 °C, 1 h). A paper frame with a circular opening (5 mm dia.) was cut and adhered to the film to define the actuating membrane shape. The frame was brush-coated with a thin film of silicone as an adhesive, positioned over the negative electrode, and cured (Figure 3(b4)).

The encapsulation method made use of the facts that PC does not mix with either PDMS or Ecoflex, and does not inhibit their curing. Uncured silicone was dispensed around the PC droplet using a syringe until it spread to cover the droplet surface; the fluid silicone formed a film that was perfectly conformal with the surface of the PC. The silicone was cured to form a polymer membrane bonded to the substrate, resulting in a sealed liquid-filled cavity with no trapped air (air can cause electric sparking and device failure, as well as loss of force). An advantage of using Ecoflex for the membrane is that it cures faster (15 min) at room temperature than PDMS (>24 h), enabling quick encapsulation of PC without exposure to heat.

### 4.2. Stacked Layout

In the stacked layout, all the layers were made from PDMS except the actuating membrane, which was made from Ecoflex. The stiffer PDMS limits deflection to the membrane area. Actuators were fabricated using a layer-by-layer lamination process, starting with creating two electrode layers and an insulating separator (Figure 3(a0)). C-PDMS electrodes (0.5 mm × 2.5 cm × 6.4 cm) were formed on glass using stencils. In the lower electrode (Figure 3(a0i)), a thinner circular stencil (0.4 mm × 1.33 cm) was placed in the center to form a reservoir. PDMS was spin coated over the slides (*t* = 1 mm) to embed the electrodes and allowed to rest at room temperature for at least one hour to allow air bubbles formed at the electrode-PDMS interface to escape. Peeling off the substrate exposed the conductive surfaces. A hole (5 mm dia.) was punched through the top electrode (Figure 3(a0iii)). An insulating PDMS layer (*t* = 0.5 mm) was formed by spin coating and manual cutting (3.8 cm × 6.4 cm), and a hole (8 mm dia.) was also punched through its center (Figure 3(a0ii)). A piece of filter paper was cut (1.27 cm dia.) to fit inside the reservoir in the bottom electrode.

The four components were then assembled (Figure 3(a1)). The bottom electrode, insulating spacer, and paper circle were exposed to oxygen plasma. The paper was placed into the reservoir, and the insulating film brought into contact with the electrode, with the 8 mm hole centered over the reservoir, sandwiching the paper between them. The top electrode was then bonded to the insulating layer using the same process, with the punched holes aligned. The electrodes extended beyond the bonded area to allow power connections.

The device was then filled and sealed within 5 min of plasma exposure. PC (0.1 mL) was added through the hole in the top electrode layer (Figure 3(a2)) until some liquid protruded. The needle was held in contact with the surface of the paper. Filling the device after surface activation ensured wetting of the silicone surfaces, and so prevented trapping of air bubbles. A circular paper ring (inner diameter (i.d.) 1.27 cm, outer diameter (o.d.) 1.52 cm) was dipped into liquid Ecoflex (<10 s), resulting in a thin film suspended by surface tension, such as seen in the formation of soap films suspended on a ring support (Figure 3(a3)). The ring was aligned with the hole in the top electrode, and the Ecoflex instantly draped itself over the PC, conforming to the shape of the droplet and forming a membrane enclosing the liquid reservoir. (Figure 3(a4)). The Ecoflex was allowed to cure at room temperature for 30 min. The final device dimensions were 5 × 50 × 100 mm^3^, and its weight was 0.012 grams (average of three devices).

The membrane thickness could be tuned by varying the thickness of the paper ring, and also by extracting the ring slowly [69]. Curing the encapsulating film at room temperature allowed time for the liquid silicone to spread and form a thin membrane with a good bond between the membrane and the substrate. (For cross-sectional images of the membrane, see the Supplementary Information.) Ecoflex 00-50 is stiffer than Ecoflex 00-30 [31], but it was used because the 00-50 bonded to PDMS while the 00-30 peeled off readily, even after plasma treatment.

### 4.3. Observations

The cutting approach to patterning is better suited to a process sequence where the application of the electrode paste and the cutting process are automated and tightly controlled. To minimize the chances of damage to the substrate, doctor-blading through stencils was used instead, which is more forgiving in a lab setting.

PDMS and Ecoflex are both cured by platinum-catalyzed crosslinking [30,31]. However, we observed differences in the bonding capabilities of these two polymers. Cured PDMS layers can be bonded to each other either by oxygen-plasma treatment or by using uncured PDMS as an adhesive. Cured Ecoflex layers can be bonded to each other or to cured PDMS only by adhering them with uncured Ecoflex or PDMS. Solidified Ecoflex could not be bonded to either solidified PDMS or Ecoflex by oxygen plasma surface treatment. In order to make Ecoflex amenable to plasma-mediated bonding, we determined that uncured Ecoflex (1:1 mixture) should be adulterated with a small amount of PDMS (10:1 mixture), using at least 1:35 by weight of PDMS:Ecoflex. Blending the two silicones can also be used to adjust material stiffness of the layers.

In order to produce hydraulic actuators by additive manufacturing, we have developed a fabrication process that involves lamination and casting of successive layers. The approach has the potential to be adapted to a roll-to-roll process, which employs lamination. Alternatively, it may be possible to produce the devices by 3D printing, which would require printed elastomers that are stretchable. Ecoflex and PDMS are not currently printable because they are two-component thermosetting polymers: solidification occurs by cross-linking catalyzed by heat. These elastomers are typically patterned by casting them, in the liquid state, over molds. Printable elastomers are stiffer and more brittle [70], and their adhesion to Ecoflex liquid for encapsulation would need to be verified. For the electrodes, the printer would need to handle a viscous nanoparticle-containing paste.

Other reported encapsulation approaches include vapor-deposition of polymers such as parylene directly on a liquid [71], and casting of UV curable polymers on a liquid [72]. In general, the encapsulation technique will be determined by the nature of the underlying substrate as well as the particular liquid. The approach used in this work is suited for the encapsulation of nonvolatile liquids in silicone devices.

After forming the electrode layer, PDMS was cast over it. The high roughness of the squeegeed surface sometimes caused air bubbles to be trapped when the PDMS was applied. Therefore, the samples were allowed to rest at room temperature to enable degassing before curing. Vacuum dessication was not applied because it led in some cases to PDMS creeping beneath the electrode layer.

It would be convenient to directly spin coat PDMS over the bottom electrode to create the insulating spacer, with the active electrode areas masked off. However, due to poor adhesion of tapes and other adhesives to silicone, the PDMS crept beneath the masks. The insulating layer therefore had to be fabricated as a free-standing film and later plasma bonded to the electrode.

Initial designs did not feature a reservoir to hold the filter paper; the paper was simply sandwiched between the layers. This created an air gap around the paper during bonding, and the trapped air could not be completely eliminated while filling the device, increasing the incidence of device failure. Due to the presence of a single port for filling the device, in order to minimize the chances of air entrapment, we introduced a reservoir with a diameter that was only slightly larger than the inlet port. To hold larger liquid volumes, for increased actuation stroke, a bigger reservoir must be incorporated. If the reservoir dimensions were increased in the plane of the device, the overall thickness of the device would be unaltered, keeping it easy to bend. However, the current design employed a single inlet port. Increasing the transverse reservoir area makes it more difficult to evacuate air trapped between the layers, near the edges of the reservoir away from the inlet port. For bubble-free fluid filling, creating a deeper, rather than wider, reservoir will minimize trapped air. Thus, the design would require optimization subject to dimensional constraints. If the design necessitated minimal device thickness, then two separate ports would be required, an inlet for filling and an outlet to allow air to escape. With a single inlet design, a syringe needle with a significantly smaller diameter than the reservoir is required, so that enough area is available for trapped air to escape. This work employed a 30 gauge needle (0.31 mm o.d.) to fill the reservoir (13.3 mm i.d.) through the port (12.7 mm i.d.). After plasma treatment, the device was filled slowly (0.1 mL/min) to allow air to escape as the reservoir was filled.

Filling the device with fluid immediately after plasma activation increases the surface energy of the silicone, which improves wetting; filling is then rapid simply by wicking, without the need for positive pressure, and the entrapped air is quickly displaced. The filling needle must be kept in contact with the paper while dispensing fluid to avoid splashing or spillage onto the surrounding silicone surface. Spillage outside the port onto the plasma-activated silicone prevents the formation of a good seal during liquid encapsulation. Although desiccation or negative pressure can be used to fill the device instead of surface activation, it increases the chances of liquid spillage due to bubbling as the air trapped in the paper and reservoir is removed.

Prior to determining polymer film casting as the most suitable method of encapsulation, we had attempted another approach to creating a sealed fluid reservoir: injection of the liquid using an ultra-fine needle, with the silicone self-sealing around the perforation after filling. As the device became thinner, it became increasingly challenging to inject liquid without piercing through it. Also, in thin devices, the silicone was unable to seal the perforation at the device operating pressure, and fluid leaked out at that site. (Another potential approach could be to fill the device with pumping liquid and freeze it in place, and then cast the membrane over the solid surface. However, PC freezes below −55 °C, at which temperature the silicones will not cure.)

The dimensions of the device layers were chosen for ease of handling during fabrication and testing. In order to decrease the size and weight of the device, the areas of the electrodes, insulating layer, and frame could be reduced significantly without affecting performance.

## 5. Results: Actuator Performance

The key metrics used to evaluate actuator performance are displacement, force, and speed. Based on these values, work, power, and energy efficiency can be determined. Before characterizing the devices, however, the adjacent and stacked designs were compared qualitatively during actuation.

### 5.1. Electrode Configuration

The first objective was to determine whether the two layouts, adjacent and stacked, both worked as intended. EO flow would be toward the negative electrode if ζ was negative for the paper/PC combination.

#### 5.1.1. Adjacent Layout

**Figure 4:**
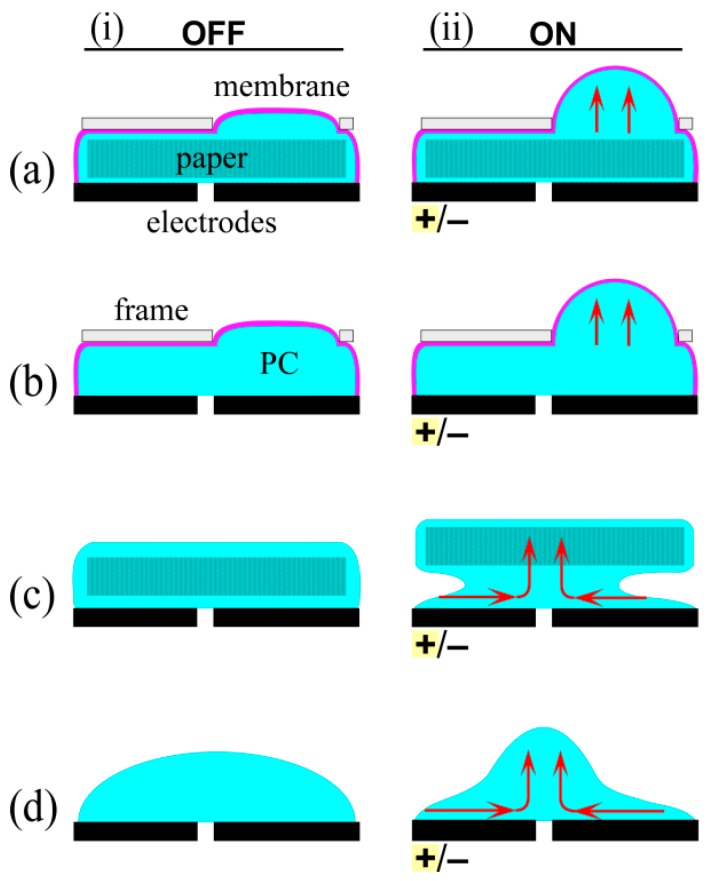
Schematic representation of fluid motion in the adjacent layout. (**ai**) The actuator comprised an actuating membrane encapsulating a pumping liquid and a paper microchannel layer above two planar electrodes; (**aii**) Application of voltage (5 kV) of either polarity caused membrane deflection; (**bi**) An actuator without the paper layer; (**bii**) Voltage of either polarity caused membrane deflection similar in magnitude to that in (a); (**ci**) An actuator without an encapsulating membrane, the liquid held by surface tension; (**cii**) Voltage of either polarity pulled the liquid between the electrodes, causing the paper to rise; (**di**) An actuator fabricated without paper and without an encapsulating membrane; (**dii**) Voltage of either polarity drew liquid toward the gap between the electrodes, forming a bulge with visible fluid circulation.

In the adjacent layout, 5 kV was applied, with the negative electrode under the membrane. Deflection was observed by eye (Figure 4a), and the membrane flattened again when the voltage was turned off (*n* = 3 devices). However, when the polarity was reversed the membrane did not further deflate, but instead bulged again. The flow was non-directional, unlike in EOF.

To investigate further, devices were fabricated with more closely spaced electrodes: the gap was reduced from 3 to 2, 1, and 0.5 mm (1 device each), and the experiment repeated. Deflection decreased with spacing, contrary to expectations for EO of increasing pressure with electric field.

To gain insight into the mechanism for the fluid flow, devices (*n* = 2) were fabricated without the paper microchannel layer (Figure 4b). The membrane deflected in the same way and to the same extent as for the previous devices, regardless of the sign of the voltage (±5 kV), and again actuation occurred for both polarities.

Open devices were fabricated (*n* = 2) having the paper but not the encapsulating membrane (Figure 4c). Upon applying voltage, again irrespective of the direction, the PC moved to the gap between the electrodes and pushed the filter paper upward.

The device was finally pared down to just a droplet of PC over the electrodes (Figure 4d). The stationary droplet had a smooth, convex surface. The voltage was ramped from zero to 5 kV. Fluid started pooling between the electrodes at 50 V. Beyond 500 V, the liquid bulge (seen by the light reflection) began moving in a circular motion between the electrodes. This behavior has previously been dubbed electrohydrodynamic circulating flow [73,74]. The fluid motion in this device was clearly not due to EO, but likely due to either the Sumoto effect [75] or the dielectric liquid bridge phenomenon [73,74,76].

#### 5.1.2. Stacked Layout

**Figure 5:**
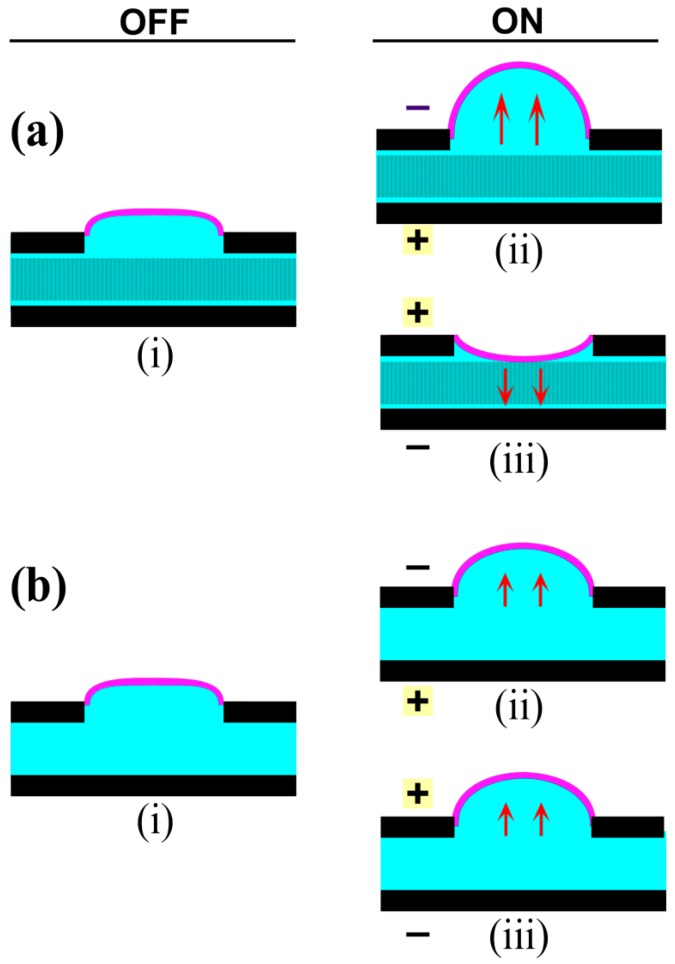
Schematic representation of fluidic actuation in the stacked layout. (**ai**) The actuator comprised an actuating membrane encapsulating the pumping fluid and a paper microchannel layer sandwiched between two electrodes; (**aii**) Application of negative voltage (100 V) to the top electrode caused membrane inflation; whereas (**aiii**) reversing polarity caused membrane depression; (**bi**) An actuator without the paper layer; (**bii**) Negative voltage on the top electrode caused membrane inflation, but to a lesser extent than in (a); as did (**biii**) the reverse polarity.

In order to force the field lines to pass through the microchannels, in the stacked layout the paper microchannel layer was sandwiched between the two electrodes (Figure 5(ai)). Fluid flow occurred from the positive to the negative electrode, consistent with a negative ζ potential, leading to membrane inflation (Figure 5(aii)). When the polarity of the field was reversed, the membrane moved downward (Figure 5(aiii)). Thus, flow in the stacked layout was directional, as expected for EO. Membrane deflection was observed beginning at 100 V, 50× lower than was required in the adjacent layout, even though the electrodes in both designs were spaced 3 mm apart. The lower operating voltage is therefore due to the face-to-face orientation of the electrodes.

A stacked layout without paper was then tested (Figure 5(bi)), in order to determine whether the flow phenomena was altered by the absence of microchannels. At 100 V, the membrane inflated irrespective of the direction of the applied field (Figure 5(bii,biii)), although the deflection was smaller than when the microchannel layer was included. Flow in this device was thus attributed to the electrohydrodynamic effects previously seen in the adjacent layout.

Based on these observations, we concluded that the stacked electrode design was better, from performance and controllability considerations, for miniature actuators. The following sections describe the performance characterization of the stacked electrode actuators only.

### 5.2. Actuator Displacement

The stroke of an actuator is its range of movement, which was taken as the membrane deflection. An inflating rubber membrane has the shape of a spherical cap [77,78], and the maximum displacement under a given pressure depends on its stiffness and diameter. The pressure depends linearly on *E* for EOF (Equation (2)), but the relationship between pressure and deflection for a rubber membrane is nonlinear [77,78].

Membrane displacement (*n* = 3 devices) was measured using a force-strain transducer in isotonic mode: a constant force of 1 ± 0.1 g was applied, and the displacement was measured over time. The positions of the force distribution plate and transducer arm were fixed as the voltages and, later, loads were varied. Voltages of 200, 400, and 600 V, with the negative potential on the top electrode, were applied, stepping on and off three times at each level. The voltage was switched after membrane deflection appeared to have stabilized. The raw data were corrected for linear drift (see the Supplementary Information).

**Figure 6:**
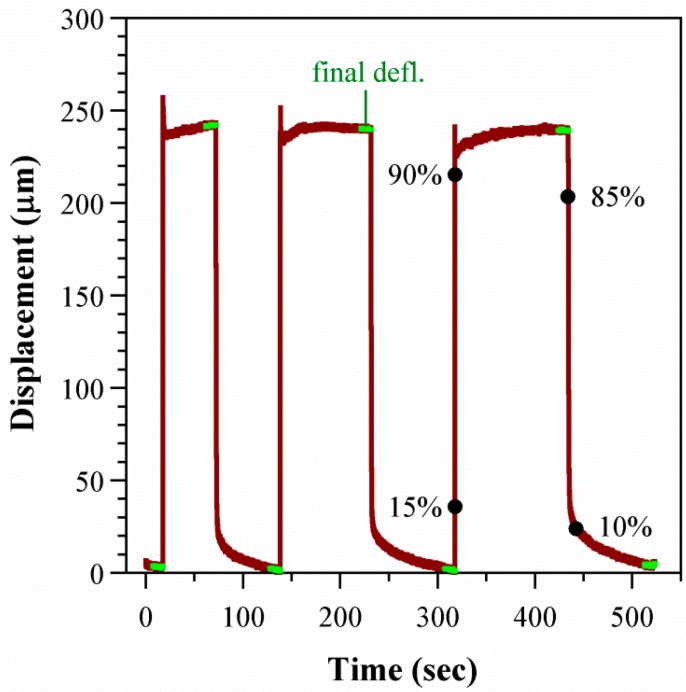
Membrane deflection over time in response to a cycle of three on/off pulses to 400 V (device 2). The points averaged to determine the deflection for each step are indicated (green), as are markers on one step illustrating the inflation values used to calculate *τ_r_* and *τ_f_*.

The time trace for one cycle in one device is shown in Figure 6. The membrane inflated quickly by hundreds of μm, with similar deflections for each cycle. Some of the deflection curves showed spikes upon membrane inflation. These occurred randomly and were not specific to a sample or voltage range.

**Figure 7:**
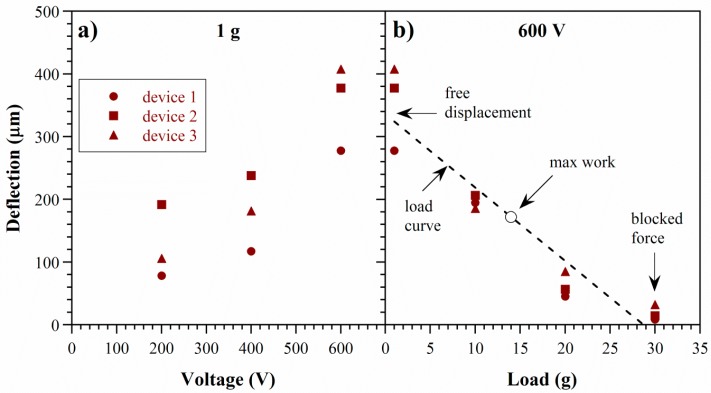
Membrane deflection in response to turning the voltage on and off. Error bars indicating the standard deviation of three runs at each voltage on the same device are smaller than the size of the data point marker. (**a**) Membrane deflection versus voltage under a 1 g load; (**b**) Load curve for the same devices at 600 V as the force on the membrane was increased. The dashed line is a linear curve fit to the average of the three devices.

The average deflection during each step was obtained from the last 10 s (last 1000 points, sampling rate 0.01 s) before the voltage was switched. The average of the three step averages is plotted as a function of voltage for three devices in Figure 7a. Deflection increased with voltage, as expected for EO. Run to run variation for a given device and voltage was ±10 μm. Between devices, the variation in deflection was larger, up to 70 μm, due to the manual fabrication and the manual positioning of the force distribution plate and transducer. The variation did not depend on voltage.

**Figure 8:**
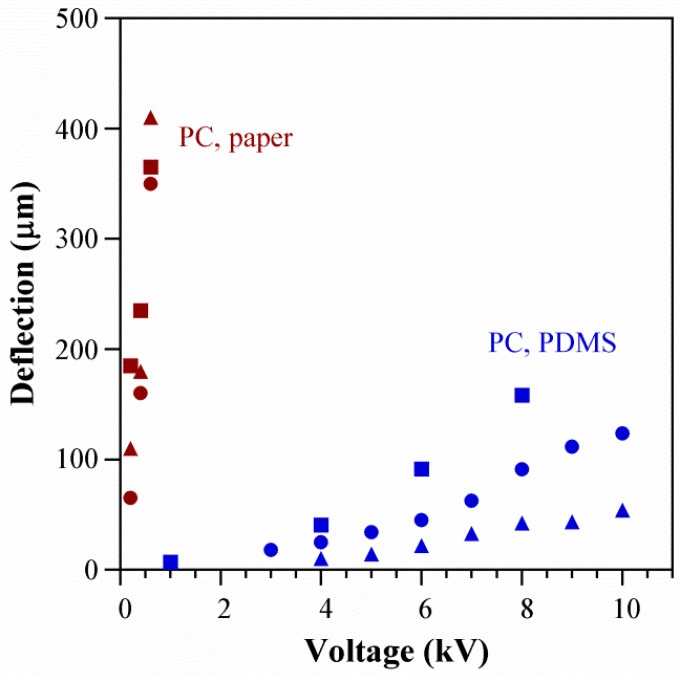
Deflection as a function of voltage in three paper-based stacked configurations (**red**) and in three PDMS microchannel devices (**blue**) [8], both filled with PC. The symbols for the stacked configuration have the same meaning as in Figure 7.

The finding that deflection scales with voltage implies that the extent of actuation can be altered using the input electrical signal, which is significant for the development of versatile control systems for smart materials and soft robotics. In principle, stroke could be increased by raising the voltage up to the dielectric breakdown field of the pumping liquid (2.2 MV/cm for PC, equivalent to 0.66 MV in these devices [79]). However, voltages above 10 kV led to immediate device failure: we observed sparking and the electrodes short-circuited, leading to noticeable Joule heating (the device became warm to the touch) as well as burn marks on the PDMS between the electrodes. In these devices, actuation was limited by the stiffness of the structure and volume of the fluid, as discussed below. For this reason and because of our interest in low-voltage operation, voltages above 600 V were not used during testing. Other methods to increase stroke might include switching to a pumping liquid with a higher dielectric limit, using microchannels with higher surface charge (zeta potential), or decreasing the membrane stiffness. Figure 8 compares the membrane deflections of the paper-based actuators to previous prototypes with microfabricated PDMS channels that had an electrode separation of 10 mm; the pumping liquid in both cases was PC and the membrane diameters 5 mm. The voltage required to achieve the same deflection in the new devices was more than an order of magnitude lower, attributed partly to the closer electrode spacing and partly to the use of Ecoflex for the membrane instead of PDMS.

Performance of the devices progressively deteriorated over time, so they were tested immediately. Throughout the day on which the device was fabricated and filled with anhydrous PC, it consistently deflected to the same extent when turned on and off. The next day, the current through the device increased by three orders of magnitude (from 0.2 to 300 mA at 600 V), and the deflection diminished. Also, electrolytic gases (bubbles) and Joule heating were observed (device warm to the touch). Two days after fabrication, EOF no longer occurred. Silicone elastomers are water permeable [80], and the encapsulated PC was thus exposed to ambient moisture, which leads to degradation [8]. Device design changes to protect the PC are discussed below.

### 5.3. Actuator Force

An actuator must be able to do more than just move: it must generate enough force to perform useful work. The measurements in the previous section represented nearly free deflection, defined as deflection under zero load. This is an actuator’s largest stroke (unless it requires pre-strain to function). To characterize force output, deflection is measured as increasingly larger loads are applied to create a “load curve”. Deflection decreases until it goes to zero at the blocked force.

To the same three devices of Section 5.2, increasing loads (1, 10, 20, and 30 g) were applied by the transducer arm, again in isotonic mode. At each value, the voltage was stepped on and off three times to 600 V. The results are shown in Figure 7b.

Deflection was reduced almost to zero at 30 g. The load curve was nearly linear and consistent across devices. Deviation from linearity is likely due to a change in contact area between the force distribution plate and the membrane at different extents of bulging. At 30 g, there is no bulging and the plate makes contact with the entirety of the membrane. This load corresponds to a pressure of 15 kPa, based on the total membrane area (20 mm^2^).

**Figure 9:**
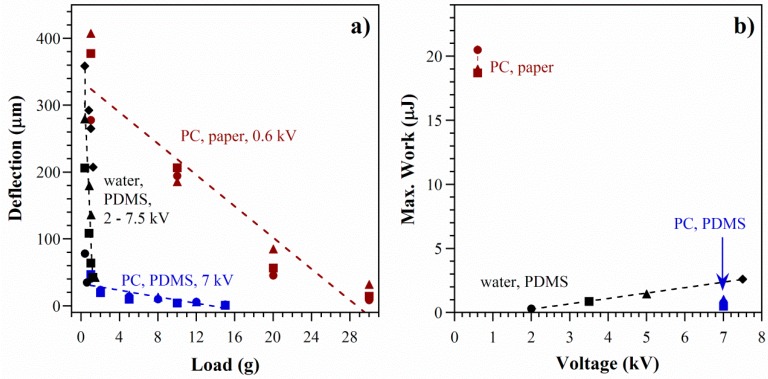
(**a**) Deflection as a function of load for the stacked configuration of this paper (same data as in Figure 7b; symbols have the same meaning) and for previously presented PDMS microchannel devices, filled with either PC (blue) or water (black); (**b**) Maximum work done by the paper-based actuator and the two PDMS devices.

Figure 9 compares the forces from the stacked paper devices with those from microfabricated PDMS devices, which had nine channels with a cross-sectional area of 150 × 40 μm^2^ and a length of 1 cm and which had been tested with both water and PC [7,8]. The stacked layout was able to generate a higher blocked force and did so at lower voltage. The free deflection was an order of magnitude larger than in PDMS channels filled with PC, presumably because the smaller channel diameters in the paper generated greater EO force (Equation (2)). This also suggests that the zeta potential in paper was substantial, even without taking steps to increase the surface charge of the cellulose pores. The large deflections in the water-filled channels [7] may have been partially due to the generation of some electrolytic gas.

The work done by an actuator is found by multiplying force by displacement. It is zero at the points of free load and blocked force and maximum at a point midway between the two. Using the deflection of 200 μm under a load of 10 g (Figure 9a), the work performed was 20 μJ. The paper-based actuators were capable of performing 10× more work at 10× lower voltage than PDMS devices with water (Figure 9b), increasing the scope for applications.

### 5.4. Actuator Speed

Speed is the third critical metric—not only must the actuator perform work, but it needs to do so in a reasonable time. The actuator speed during inflation is determined by the EO flow rate, while during deflation it is determined by the membrane stiffness, which provides the back-pressure.

**Figure 10:**
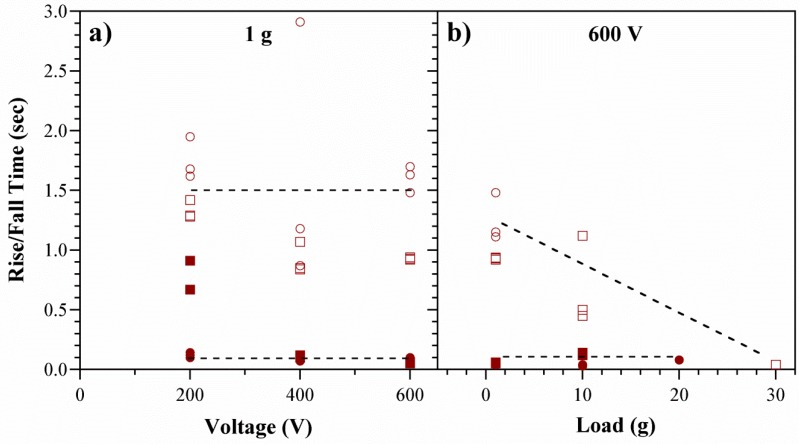
Rise (filled symbols) and fall (open symbols) times for two devices (1 and 2). Points represent individual on/off steps, three per device. (**a**) Times as a function of voltage under minimal load; (**b**) Times at a fixed voltage as a function of externally applied load.

The deflection versus time curves obtained during the above experiments were evaluated. The rise time *τ_r_* was defined as the time it took to go from 15% to 90% of the average deflection, and the fall time *τ_f_* from 85% back down to 10% (see Figure 6). The devices inflated quickly by EOF (*τ_r_* = 0.1 s) and deflated more slowly (*τ_f_* = 1.5 s) (Figure 10a). Increasing the voltage did not significantly alter the rise or fall times. Figure 10b illustrates that *τ_r_* was unaffected by external loads. In contrast, *τ_f_* decreased with load. This was expected due to the increased force on the membrane, raising the back-pressure.

In the PDMS microchannel device, a break-in phenomenon was observed [8]: membrane inflation took longer during the first actuation cycle, but successive inflations were faster. This did not occur in the paper-based devices: the inflation time for the first cycle was similar to that in successive cycles.

Efficiency is defined as the work performed divided by the energy input. Under a load of 1 g, the current through the device at 600 V was 0.18 mA (refer to the Supplementary Materials for further information). For a rise time of 0.1 s, the energy input was 11 mJ. Using a work of 20 μJ from above yields an efficiency of 0.2%.

**Figure 11:**
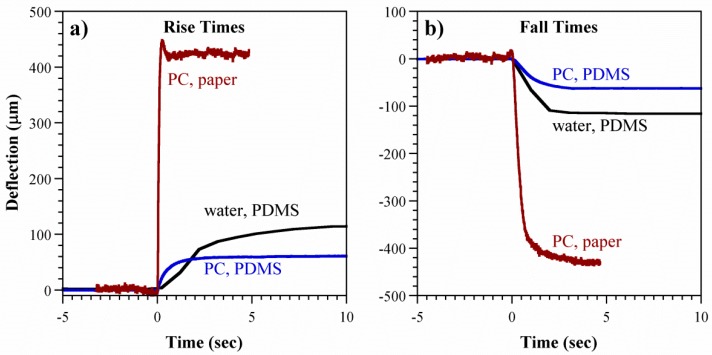
Comparison of deflection-time curves in the stacked configuration and in PDMS microchannel devices. (**a**) Rise times and (**b**) fall times with starting deflections set to zero to allow comparison. (Conditions: load = 1 g; paper/PC, 200 V; PDMS/PC, 7 kV; PDMS/water, 3.5 kV, but times are not voltage dependent).

**Figure 12:**
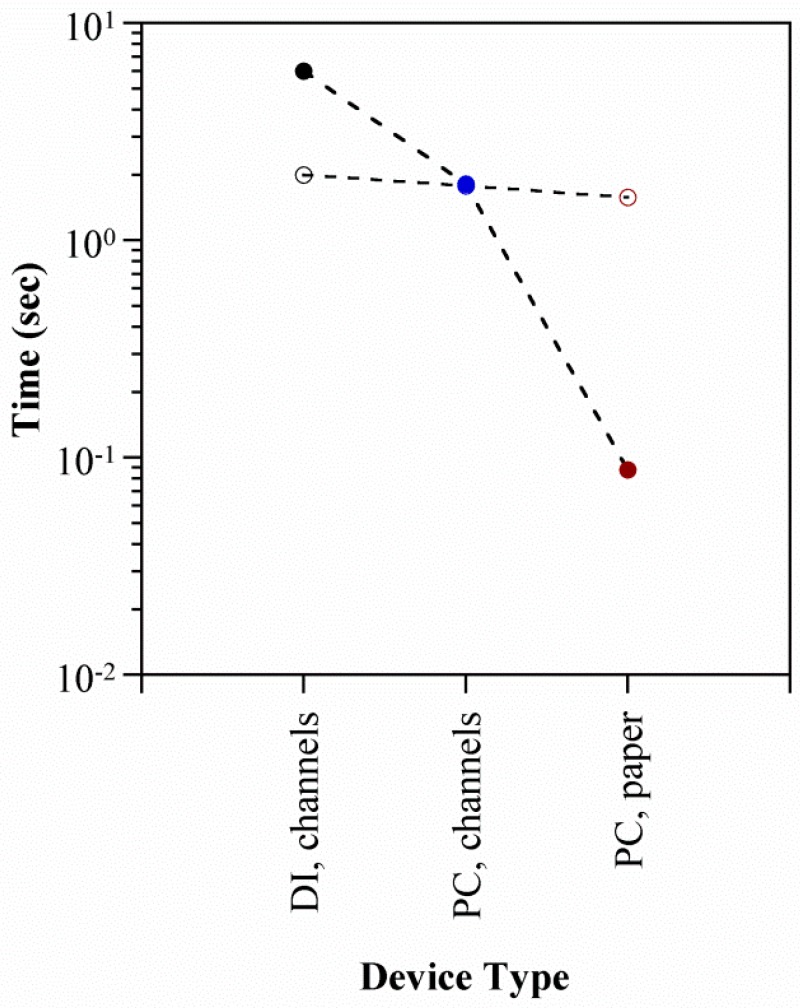
Summary of actuator response times in the paper-based stacked devices and previous microchannel devices using PC and DI water. Filled circles represent inflation and open circles deflation.

The rise and fall curves are compared with those in the PDMS microchannel devices in Figure 11, and the actuation times are summarized in Figure 12. The points in the latter represent the average (all voltages) *τ_r_* (solid markers) and *τ_f_* (open markers) for each device type. Inflation was substantially faster in the paper-based device, with *τ_r_* an order of magnitude smaller. This is attributed to the larger cross-sectional area *A_p_* available for electroosmotic pumping (Equation (1)). Assuming a 50% porosity in the paper, *A_p_* = 0.6 cm^2^ compared to 5 × 10^−4^ cm^2^ for the previous microchannel devices. Deflation speeds, on the other hand, were similar. This was expected based on the physics of the restorative pressure-driven back-flow, since the membranes were of comparable stiffness (refer to the supplementary for further information).

## 6. Discussion

In this work, we have solved some of the principal challenges in developing microfluidic actuators. The external pump was eliminated by employing an electrically-driven pumping mechanism. The protruding fluidic tubes typically required for filling were abolished by employing a new fluid encapsulation process. The voltage was lowered by employing paper for the pumping surface, which also yielded a larger cross-sectional area and thus a greater flow rate. The force was raised by swapping PC for water as the fluid.

Additional steps can be taken to further enhance device performance in future work. Increasing actuator stroke is of immediate concern. The liquid is incompressible; therefore, expansion in one part of the structure can only be achieved by collapse of another region. Currently, expansion occurs at the more compliant Ecoflex membrane, while the stiffer PDMS and paper provide structural support and confine the actuation location. Whereas the previous PDMS prototype included a second, collapsible reservoir, this device did not. The membrane displacement could be raised by further lowering the stiffness of the reservoir’s silicone base layer, already reduced in thickness under the paper, allowing it to deflect upward more easily, or by other features that allow structural crumpling.

Pumping force can theoretically (Equations (1) and (2)) be increased by boosting the surface charge on the paper via chemical functionalization: highly charged groups can be added via polyelectrolyte deposition [81] or silane treatment [82]. Alternately, a different porous material with a higher surface charge and smaller pores can be employed instead. For example, we have observed EOF using sheets of spun polyethylene, cotton, polyurethane sponges, and glass microfiber filters in place of the paper. Fabrics may also be considered, which would improve the mechanical flexibility of the device.

Encapsulation prevents evaporative loss of the pumping fluid, protects the pumping fluid from external contamination (for PC, ambient moisture [8,83]), and keeps the pumping fluid from making contact with the external environment (e.g., microfluidic samples, biological tissue). Silicones swell in many liquids [80] and are permeable to gases. The challenge is thus to devise a stretchable layer that is impermeable. One possibility would be to coat the device with an inorganic layer, such as a metal or oxide. To allow the non-stretchable bilayer to inflate, one could employ wrinkles [84]. The membrane would be inflated using a mechanical pre-load or by actuation, and coated in the expanded state. Subsequent collapse of the membrane would lead to wrinkling, maintaining continuity of the inorganic layer. With PC, the coating would need be deposited below 116 °C (PC flash point, [85]).

Autonomous devices that do not require an external high voltage power supply would be advantageous in some applications. To permit device operation with an on-board battery, as well as integrated circuit control, the voltage requirement must be reduced still further. One straightforward method is to decrease the distance between the electrodes. Ideally, the channel layer between them would therefore be thin, in addition to having a large surface area, high porosity, and high charge density.

It is desirable to use a highly conductive electrode material in order reduce Joule heating and power consumption. The loading of conductive particles should therefore be well above the percolation threshold. There was no visual evidence of electrode damage after normal use, although additional work would be required to confirm that the electrodes do not degrade under extended periods of EO. In battery applications, PC is used as a supporting electrolyte for lithium and sodium. In the presence of these ions, there can be changes in the graphite anode surface when the PC undergoes electrochemical decomposition to form gases at the electrode–PC interface [86].

Our fabrication process yielded a single, self-contained actuator unit. The process could be further developed to combine multiple actuators in series and parallel to form arrays, stacks, and complex multi-dimensional moving structures. Units could be joined in multiple orientations by plasma bonding, magnets, or adhesive. Electrical connections could be made with the conductive carbon composite, which can be easily applied and patterned. Composite structures employing materials with different stiffnesses would enable programming of the directionality of actuation.

The actuator developed in this work is thin and therefore rollable. This technology could therefore be useful for biomedical applications such as in an adjustable stent or artificial sphincter to control the flow of body fluids. A preliminary flow control test setup is described in the Supporting Information. Other applications of these actuators may be in miniature lenses, integrated pumps and valves for lab-on-a-chip microfluidics, soft robotic limbs and joints, and elastomeric surfaces that can change color or texture in response to an electrical stimulus.

## 7. Summary and Conclusions

Flexible devices were fabricated using layer-by-layer assembly of parts composed of silicone and paper. Electrodes were made of a conductive composite of carbon nanoparticles in silicone, and a porous paper pumping layer was sandwiched between them. After filling the device with pumping fluid, it was encapsulated with a film of liquid silicone that cured conformally over the fluid. The silicone skin then served as the actuating membrane.

Electroosmotic flow in the device utilized the pores in the paper as microchannels and the cellulose fibers as the charged walls. Another configuration with the electrodes adjacent to each other under the layer of paper, although having a simpler fabrication process, did not result in EO, but in other electrokinetic motions. The device design must enforce only a single fluid path, through the paper, in order to achieve the desired pumping.

Actuator strokes of up to 400 μm and blocked forces of 30 g were achieved at only 600 V. Actuation was quick, taking less than 0.1 s. The performance was substantially improved over devices utilizing microchannels formed by soft lithography. Further improvements could be achieved in future work by modifications such as increasing the surface charge on the paper, adding a water barrier, and introducing collapsibility to the structure.

## Supplementary Materials

The supplementary materials are available online at www.mdpi.com/2073-4360/8/11/400/s1. They include further information on the formulation and properties of the carbon electrodes; photographs of the devices during fabrication; details of how the deflection data were collected and processed; further information on membrane formation and properties; exploration of the use of the device for load sensing; and operating the device in a rolled configuration for potential application for flow control.
