# Supplementary material for: Fabrication of a Miniature Paper-Based Electroosmotic Actuator
Source: Polymers (Basel). 2016 Nov 15;8(11):400. doi: 10.3390/polym8110400 (PMC6432343; doi:10.3390/polym8110400)
Supplement: Supplementary file 1 [file polymers-08-00400-s001.pdf]

Supplementary Materials: Fabrication of a Miniature Paper-Based Electroosmotic Actuator

Deepa Sritharan and Elisabeth Smela

1. Carbon Electrodes

Hexane lowers the viscosity of the uncured elastomer/carbon mixture, making it easier to process, but it lowers conductivity, leads to brittleness, and results in small holes [62]. The role of hexane was therefore investigated. The two elastomers also needed to be compared as hosts. For this work, Ecoflex 00-30 was used rather than 00-50 because it is softer.

PDMS-based films with 14 wt % carbon black were formed with varying amounts of hexane (Sigma-Aldrich) to determine the effect of the solvent on electrical resistance. Five formulations were prepared with hexane at 0, 100%, 150%, 200%, and 300% by weight, relative to the pre-polymer. Without hexane, the suspension was a thick paste that had to be applied by doctor blading; with hexane, the suspensions could be cast. These films were poured to a thickness 1 mm, although as they dried the samples with higher hexane fractions were thinner because the volatile hexane escaped. Four films of each concentration were formed on glass slides; 5 g of each mixture was deposited on the glass, and cured in a conventional oven for 2 h at 65 °C, at which time they were dry to the touch.

Film resistance was measured using a multimeter (Fluke 179) at five locations on each sample, giving a total of 20 measurements at each hexane concentration. Electrical contact was made by manually applying the probe tips to the surface at a separation of 1 to 3 cm. The resistance was measured without removing the films from the substrates the day after they were cured (Figure S1).

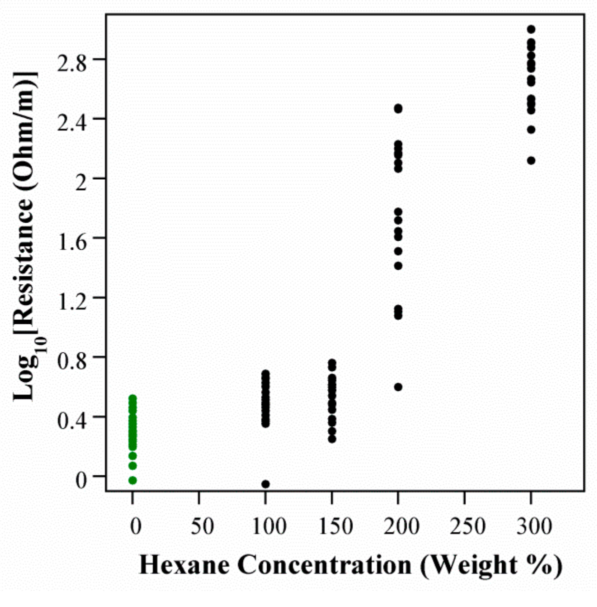

Figure S1. Resistance of cured PDMS with 14 wt % carbon black (log scale) as a function of the amount of hexane added to the prepolymer. Each point represents a single measurement (4 samples, 5 pts each).

The resistance increased with hexane concentration, despite the fact that the hexane was expected to have evaporated fully. When the measurements were repeated again later (≥24 h) the resistance did not change noticeably. There was significant sample-to-sample variation, but those samples produced without hexane had an average resistance of 0.2 kΩ/cm, while those produced with 300% hexane had an average resistance of 50 kΩ/cm, a factor of 250 greater.

Visual observation of the films revealed that the homogeneity of the cured films decreased with hexane concentration. The carbon aggregated in some areas and cracks appeared. At several

locations it was evident by inspection that the nanoparticles had settled at the bottom of the film, while the polymer formed an insulting layer on top. These issues were not observed in the films prepared without hexane.

Furthermore, hexane led to embrittlement. The samples were released from the glass; their edges were trimmed using a razor blade to just less than 5 cm × 7.5 cm, and the films were peeled off the glass slide slowly, taking care to minimize stretching of the film. The ends of the films were clamped (binder clips). One end was fixed and the other was stretched by hand over a ruler. The samples containing more than 200% hexane tore when the films were peeled from the glass. Samples with 100% and 150% tore at 20% strain. Therefore the use of hexane for electrode preparation was minimized.

Films were produced without hexane, again at 14 wt %, with the two polymer hosts, PDMS and Ecoflex. The resistance was measured at various fixed strains, applied as described above, to determine the extent to which the electrodes could be stretched and how strain affected the conductivity. Measurements were made when the electrodes were still attached to the glass (0% strain), after peeling off the glass (assumed 1% for plotting purposes), and at 10% to 250% strains.

The Ecoflex outperformed the PDMS on every measure (Figure S2). The Ecoflex electrodes had lower resistance, the resistance varied less with strain, at a fixed strain the resistance among samples was more consistent, and they had a higher strain to failure. The carbon-PDMS films tore at strains above 200%, and carbon-Ecoflex films failed at 300% strain.

Despite these advantages, we used PDMS for the electrodes because of one critical step in the fabrication process: oxygen plasma bonding of the layers to create thin devices. Plasma surface treatment is an effective method of bonding PDMS to PDMS, without the application of additional layers of adhesive. It is not effective with Ecoflex.

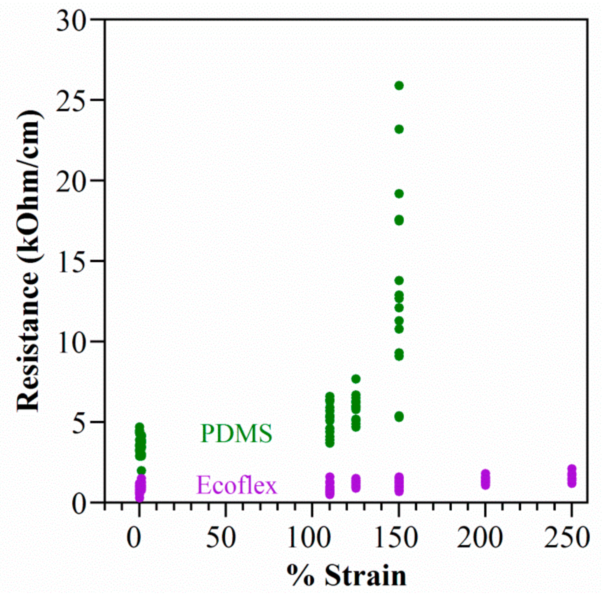

Figure S2. Resistance of carbon-PDMS and carbon-Ecoflex under strain. Each point represents a single measurement (4 samples, 5 pts each).

2. Fabrication

Figure S3 shows various stages during fabrication.

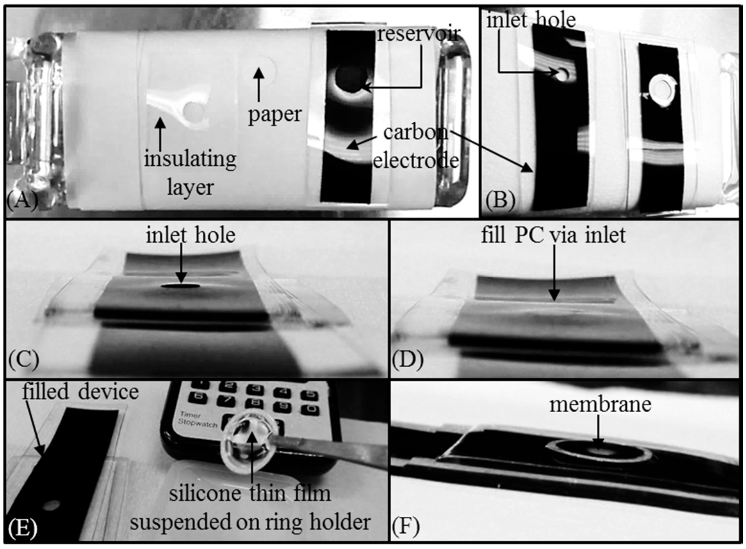

Figure S3. (**A**) The insulating layer, filter paper and positive electrode were exposed to plasma. The filter paper was placed in the reservoir. The insulating layer was then adhered to the electrode, sandwiching the paper between them; (**B**) The negative electrode was plasma bonded on top of the insulating layer; (**C**,**D**) The device was filled with PC through the inlet hole in the negative electrode; (**E**) A ring was dipped in liquid Ecoflex silicone to extract a thin film. The liquid film was placed over the inlet hole filled with PC and allowed to cure to form the final encapsulated device; (**F**) The completed device.

3. Deflection Data

The data shown in Figure 6 in the main paper were obtained by processing the raw deflection (Figure S4) from the force-strain transducer (Figure S5), for which upward motion corresponded to smaller values. First, drift was subtracted: a straight line across the entire cycle was calculated using the start and end points of the run (3 cycles). The curve was then flipped vertically, so that increasing displacement corresponded to inflation. Finally, the baseline offset at the start of the first cycle was subtracted to set to it to zero.

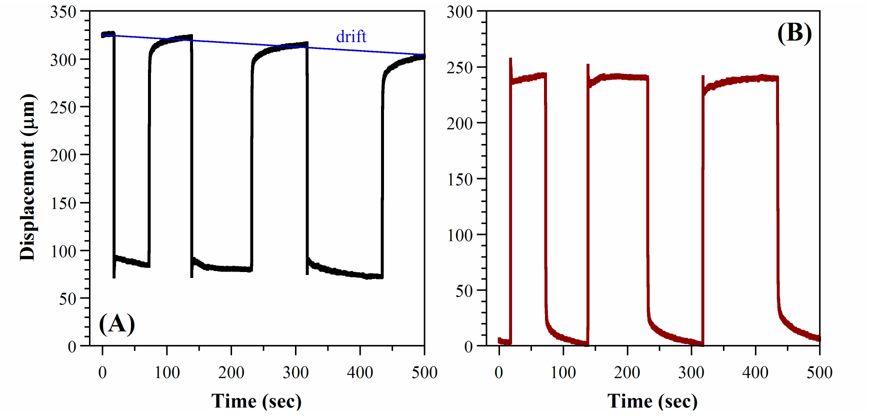

Figure S4. Data processing of deflection data collected over time using the force-strain transducer. The data presented in Figure 6 are shown again in (**B**); They were obtained by processing the raw data shown in (**A**), removing the linear drift, inverting the *y*-axis to put rise and fall in the expected up/down orientations, and setting the baseline at the start of the experiment to zero.

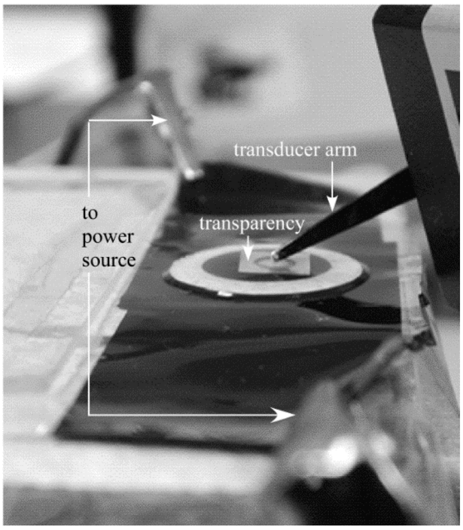

Figure S5. Experimental set up for measuring deflection or force.

4. Membrane Properties

Figure S6 shows a cross-section of a membrane, sectioned using a razor blade, from a stackedlayout actuator fabricated by film casting (Section 4.2). The membrane was thicker in the center (270 ± 36 μm, average of three samples) than at the periphery alongside the walls of the reservoir (168 ± 22 μm), although while casting the liquid Ecoflex film it initially appeared (by visual inspection) to be thinner at the center of the draped droplet. This membrane shape may develop as the film stabilizes after casting, during which a concave meniscus is formed, causing the Ecoflex to flow towards the center of the meniscus. The overall thickness is governed by the thickness of the ring that supports the film (190 μm).

In Section 5.4 the flattening speeds of the various actuators were the same when the voltage was turned off. This is because their stiffnesses were similar. The reported elastic modulus of PDMS is 750 kPa [80], and that of Ecoflex is an order of magnitude smaller at 83 kPa [81]. In the previous microfluidic device [25] the PDMS membranes were 25 μm thick, and in the stacked paper-based device the Ecoflex membranes were an order of magnitude thicker, on average 220 μm. The stiffnesses, given by the product of thickness × modulus, were thus nearly equal at 18.8 and 18.3 N/m, respectively. To produce a thinner membrane, strategies may include decreasing the thickness of the ring and pre-bulging the droplet by applying an upward force at the base of the reservoir.

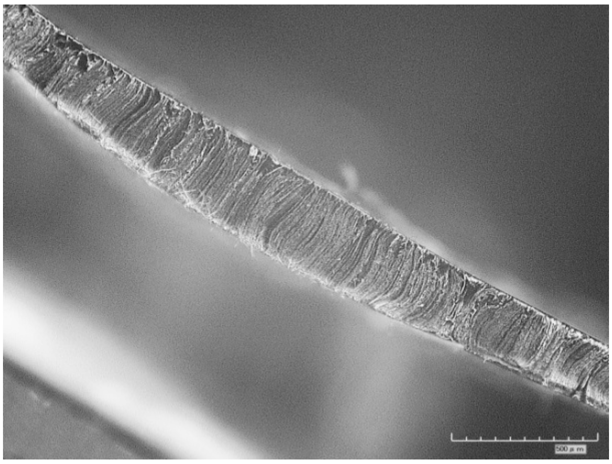

Figure S6. Micrograph of membrane cross section. The scale bar is 500 μm long.

5. Inverse Effect—Load Sensing

The current-voltage characteristics of the device were measured using a source-measure unit (Keithley 237). The instrument was programmed to source voltage and measure current simultaneously and continuously by a Matlab code with the Measurement and Automation Explorer module (National Instruments); a USB-to-GPIB adapter was used to connect the source-measure unit to the computer. Two devices were tested on two consecutive days. The current through the device was also measured with external loads applied on the membrane. Force was applied to the membrane by placing weights on top (Figure S7); a small piece of Kimwipe was placed between the membrane and weights to prevent stiction. The voltage sweep was repeated as the load was applied.

In order to determine the efficiency of the paper-based EOF actuator, the current-voltage characteristics were recorded ("0 g" curve). Two devices (1 and 2 in the main text) were tested. The voltage was increased in a linear sweep from 300 to 1100 V. The current changed proportionally as the voltage increased. The results from device 1 are shown in Figure S8.

When weight was added (15–45 g curves), the current decreased, with the *I-V* curves shifting downward with increasing load. However, when the load was removed ("0 g, unloaded") the current was not restored (within 5 min of unloading), but remained the same as it was under 45 g. The unloaded device was allowed to rest for 30 min to allow the fluid pressure to equilibrate and the elastomers to relax. The position of the *I-V* curve after this ("0 g, unloaded, relaxed") was higher, approaching the starting position.

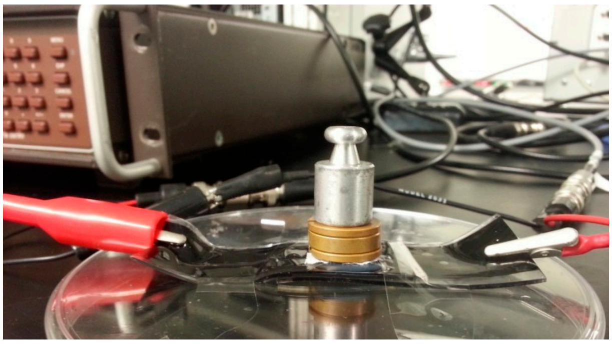

Figure S7. Experimental setup for studying the *I-V* characteristics of the paper-based stacked-layout electroosmotic actuator. The electrodes were connected to a sourcemeasurement unit. The current was recorded as a function of applied voltage, and the influence of applied loads on current was studied.

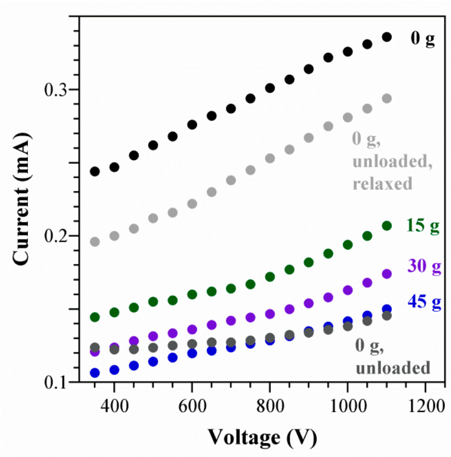

Figure S8. *I-V* curves recorded under applied loads of 0, 15, 30, 45, 0, and 0 grams (in that order) as the voltage was ramped linearly from 350 to 1100 V. The 45 gram weight was removed and a measurement was done at 0 g within 5 minutes (0 g, unloaded). After 30 min the 0 g measurement was repeated (0 g, unloaded, relaxed).

6. Flow Control

A single device was rolled and placed inside a rigid tube (inside a section of a 3 mL syringe barrel, diameter 8.6 mm), with the electrodes extending outside the tube connected to the actuator power supply (Figure S9A). Two 1 mL syringes were connected to either end of the central tube holding the device. Using a syringe pump a flow rate of 0.1 mL/s was applied (setup shown in Figure S9B). When voltage was applied to the actuator minimal alteration of flow was observed. This was likely because the magnitude of actuator stroke (400 μm was much smaller in comparison to the size of the tube (8.6 mm) (depicted in Figure S9C). Future work will therefore involve optimization of the EO valve dimensions with respect to the flow conduit geometry, and combining multiple actuating units (cross section of smaller flow tube with four actuator units shown in Figure S9D, in order to achieve flow control.

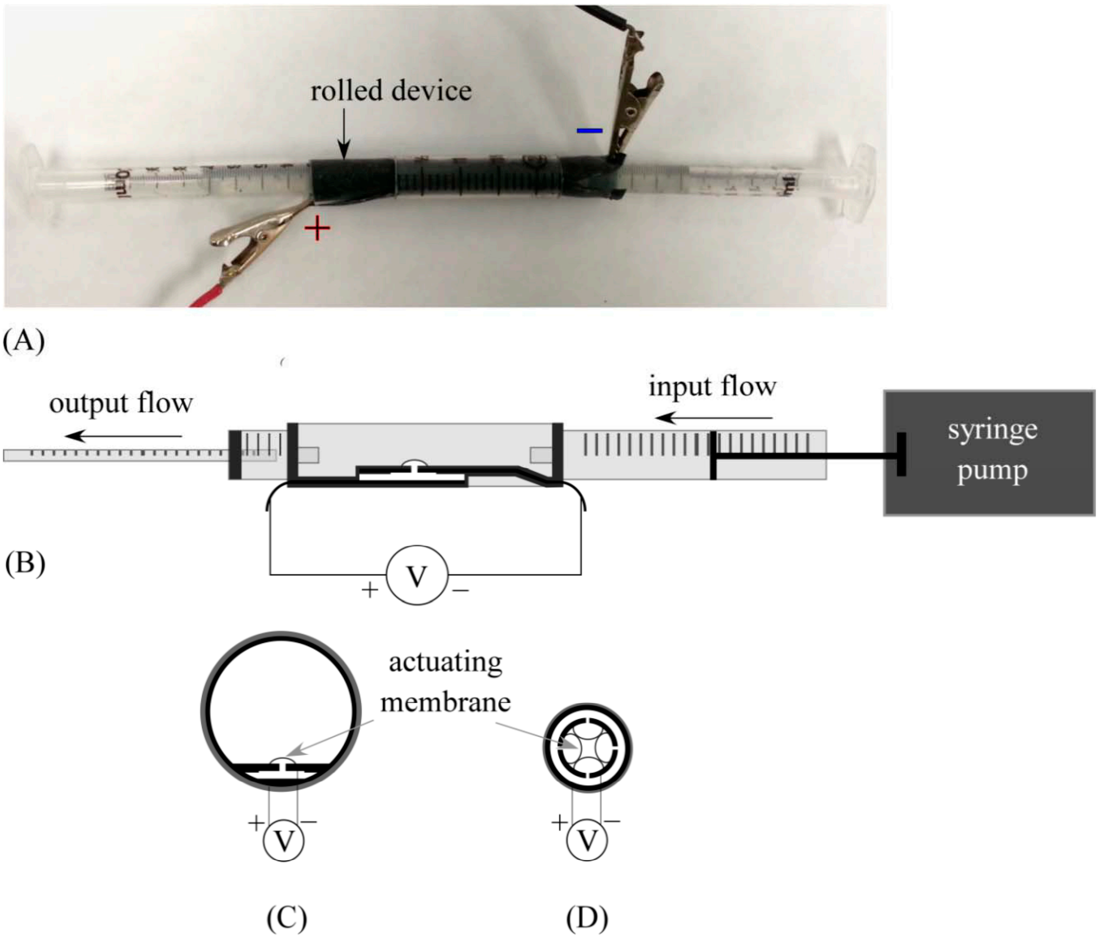

Figure S9. Schematic of flow control using EO actuation. (**A**) Photograph of device rolled and placed in a flow channel; (**B**) Transverse view of setup with a valve comprised of a single actuator, placed inside a flow tube; (**C**) Cross-section of the tube containing a single actuator; (**D**) Cross-section of proposed design: the valve size, and number of actuating membranes must be optimized relative to flow conduit size in order to modulate the effective cross-sectional area for flow.
